# Therapeutic Drug Monitoring of Ceftazidime-Avibactam Concentrations in Carbapenem-Resistant *K. pneumoniae-*Infected Patients With Different Kidney Statuses

**DOI:** 10.3389/fphar.2022.780991

**Published:** 2022-06-22

**Authors:** Xin-Qi Teng, Qiang Qu, Yue Luo, Wen-Ming Long, Hai-Hui Zhuang, Jiao-Hua Xu, Yu-Xin Wen, Hui-Lin Zhang, Jian Qu

**Affiliations:** ^1^ Department of Pharmacy, The Second Xiangya Hospital, Central South University, Institute of Clinical Pharmacy, Central South University, Changsha, China; ^2^ Department of Pharmacy, Xiangya Hospital, Central South University, Changsha, China; ^3^ National Clinical Research Center for Geriatric Disorders, Xiangya Hospital, Central South University, Changsha, China; ^4^ Department of Pharmacy, The People’s Hospital of Liuyang, Liuyang, China; ^5^ Department of Pharmacy, Second People’s Hospital of Huaihua, Huaihua, China; ^6^ Department of Pharmacy, The Fourth People’s Hospital of Yiyang, Yiyang, China; ^7^ Department of Pharmacy, Lixian People’s Hospital, Lixian, China; ^8^ Department of Pharmacy, Lixian Hospital of Traditional Chinese Medicine, Changde, China

**Keywords:** carbapenem-resistant K. pneumonia, therapeutic drug monitoring, KPC, ceftazidime-avibactam, CAZ-AVI

## Abstract

**Aims:** Carbapenem-resistant *K. pneumoniae* (CRKP) is the most common carbapenem-resistant *Enterobacteriaceae* with high mortality. Ceftazidime-avibactam (CAZ-AVI) has exhibited excellent *in vitro* activity *in vivo* against CRKP. However, the efficacy of CAZ-AVI in KPC-producing CRKP-infected patients with different kidney statuses varies, such as renal insufficiency, normal renal function, and augmented renal clearance (ARC). We explored the use of therapeutic drug monitoring (TDM) to evaluate the concentration and efficacy of CAZ-AVI in CRKP-infected patients with different kidney statuses.

**Methods:** Serum concentrations for CAZ and AVI were determined by the high-performance liquid chromatography method. Bacterial identification, routine susceptibility testing, renal function index, and others were performed in standard protocols in the hospital’s clinical laboratories.

**Results:** In the two patients with ARC, in case 1, CAZ-AVI 2.5g q6h was used with good efficacy, and the concentrations were up to the pharmacokinetics/pharmacodynamics targets. In Case 2, 2.5 g q8h was used with invalid effectiveness, and AVI C_min_ was only 0.797 mg/l, which is lower than the PK/PD target. Case 3 was renal insufficiency using CAZ-AVI 1.25 q8h, and case 4 was normal renal function using 2.5 g q8h. Their concentrations were both up to the PK/PD targets.

**Conclusion:** TDM results demonstrated that CAZ-AVI steady-state plasma concentration varies among patients with different kidney statuses, providing evidence for the utility of TDM of CAZ-AVI in individualized drug dose adjustment. ARC patients may need more CAZ-AVI daily doses than the standard dose.

## Introduction

World Health Organization lists carbapenem-resistant Enterobacteriaceae (CRE) as the “critical” group of bacteria that pose the greatest threat to human health (WHO, 2017). Especially, carbapenem-resistant *K. pneumoniae* (CRKP) is also the most common CRE with high mortality (Zhang et al., 2018).

Carbapenem resistance within *Enterobacteriales* derives from two main mechanisms, including the acquisition of carbapenemase genes that encode enzymes capable of hydrolyzing carbapenems and a decrease in the uptake of antibiotics by a qualitative or/and quantitative deficiency of porin expression in association with overexpression of β-lactamases that possess a very weak affinity for carbapenems (Nordmann et al., 2012). Carbapenem-hydrolyzing β-lactamases are of three categories: 1) class A serine enzymes, such as KPC-type enzymes; 2) class B metal enzymes, such as NDM, VIM, and IMP metallo-β-lactamases, and 3) class D serine enzymes, such as OXA-48–type enzymes, which confer resistance to most β-lactams, including carbapenems (Usman Qamar et al., 2020; Findlay et al., 2021). KPC-Kp exhibits extensive drug-resistant phenotypes, and there has been an ongoing debate on the best strategies for managing KPC-Kp infections.

Avibactam is a novel non-β-lactamβ-lactamase inhibitor that restores the activity of ceftazidime against those producing KPC and OXA-48 *carbapenemases* (Chen et al., 2021). Ceftazidime/avibactam (CAZ-AVI) has exhibited excellent *in vitro* activity *in vivo* against KPC-producing *Enterobacteriaceae* and superior efficacy against these isolates compared with other established treatment regimens (van Duin and Bonomo, 2016; van Duin et al., 2018; Tumbarello et al., 2021).

However, the efficacy of CAZ-AVI in KPC-producing CRKP-infected patients with different kidney statuses varies. The kidneys predominantly clear CAZ and AVI with similar pharmacokinetics (PK) profiles and protein binding (Li et al., 2020). Therefore, patients with different renal function state, such as renal insufficiency, normal renal function, and augmented renal clearance (ARC), have different degrees of drug clearance (Li et al., 2020).

We report a case series about KPC-producing CRKP-infected patients with different kidney statuses treated with CAZ-AVI. Therapeutic drug monitoring (TDM) evaluated the adequacy of therapy and guided the dose selection and dose optimization for CAZ-AVI.

## Materials and Methods

### Sample Collection and Therapeutic Drug Monitoring

Serum samples were planned at the following intervals 3 days after CAZ-AVI treatment: 0.5 h immediately before the next dose and 5 min after intravenous drip. Blood samples were collected and were then immediately sent to the central laboratory for centrifuging and serum separation. Serum samples were stored in transport tubes at −80°C until they were assayed. The high-performance liquid chromatography (HPLC) method determined serum concentrations for CAZ and AVI referring to relevant literature studies (Wang et al., 2019; Deng Yang et al., 2020). Plasma concentrations were determined by HPLC on CAPCELL PAK C18 column (4.6 mm × 250 mm, 5.0 μm) with mobile phase A of 50 mmol L^−1^ potassium dihydrogen phosphate solution, mobile phase B of the mixed organic phase (acetonitrile/methanol/water = 7∶2∶1), A/B (V/V, 93∶7), a flow rate of 1.0 ML min^−1^, and a wavelength is 254 nm.

### Antimicrobial Susceptibility Testing

Bacterial identification and routine susceptibility testing were performed in standard protocols in the hospital’s clinical laboratories. Susceptibilities were retested using the VITEKVR 2 system (bioMe´rieux, Marcy-l’E′ toile, France). The MICs of CZA for each strain were evaluated using the trace broth double dilution method. A fixed avibactam concentration of 4 μg/ml in conjunction with two-fold dilutions of ceftazidime was used in these tests according to Clinical and Laboratory Standards Institute (CLSI) guidelines (Institute, 2020). Results were interpreted by CLSI guidelines (Institute, 2020). The modified Hodge test (MHT) with EDTA disk test was used to detect carbapenemase-producing *Enterobacteriaceae* phenotypically (Yan et al., 2014).

## Case Presentation

### Case 1

A 56-year-old man was admitted to the Neurological Intensive Care Unit (NICU) due to fever for 3 weeks, and abnormal mental behavior for 2 weeks. The patient was admitted with a temperature of 39.3°, accompanied by chills, fatigue, and head pain. Head MRI showed abnormal signals in the left hippocampus, and the CT scan of the lungs showed lung infection. Considering the possibility of viral meningoencephalitis and pneumonia, meropenem 1g q8h, acyclovir, and foscarnet sodium were used for antiviral and antibacterial purposes. The patient was lethargic. Peripherally inserted central catheter and ventilator were used in the patient. The creatinine clearance rate (CrCl) was 145.32 ml/min/1.73 m^2^. On the third day in the hospital, bilateral blood cultures showed ESBL (-), carbapenem-sensitive pneumonia. The sputum culture showed *stenotrophomonas maltophilia* and hence SMZco was added. On day 11, bilateral blood cultures turn negative. While on day 15, the urine culture revealed the CRKP (800 cfu/ml) urinary tract infection, only sensitive to polymyxin B (PMB) (MIC ≤ 1) and CAZ-AVI(MIC = 2/4). On day 21, CRKP strains were isolated from sputum and bronchoalveolar fluid (BALF) cultures, only sensitive to PMB (MIC ≤ 1) and CAZ-AVI (MIC = 2/4). Then, the antibiotic regimens were changed to meropenem 2g q8h plus fosfomycin 4g q8h. Subsequently, CRKP was repeatedly detected in sputum, alveolar lavage fluid, and urine. While the clinical efficacy was not satisfactory, considering that the CrCl was 194.98 ml/min/1.73 m^2^, CAZ-AVI 2.5g q6h was substituted for fosfomycin on day 25. Three days later, TDM of the concentration of CAZ-AVI showed the following: CAZ C_max_ 149.21 mg/L and C_min_ 14.33 mg/L; AVI C_max_ 37.76 mg/L, and C_min_ 3.16 mg/L. After 10 days of CAZ-AVI treatment, the sputum and urine cultures turned negative. Five more days later, the patient’s bacterial infection condition improved, and the antibiotic regimens were discontinued. On day 58, the patient’s intracranial condition was stable and he was transferred to a local hospital for rehabilitation.

### Case 2

A 32-year-old man with cerebellar infarction and bodyweight of 80 kg was transferred to NICU after the venous embolus, posterior fossa decompression, dilated dural repair, and pulmonary infection on May 18, 2021. Upon admission, the patient had a temperature of 39°C, a pulse of 140 beats/min, and was delirious, with a white blood cell count of 16.25 × 10^9^/L, a neutrophil ratio of 91.60%, the C-reactive protein of 278.00 mg/L, the procalcitonin of 0.321 ng/ml, and t interleukin-6 of 6137.00 pg/ml. The initial anti-infection regimen was meropenem (2g q8h) combined with linezolid (600mg q12h). On day 16, CRKP was isolated from the cerebrospinal fluid and sputum, both sensitive to PMB (MIC ≤ 1) and CAZ-AVI (MIC = 2/4). PMB was administered intravenously (75mg q12h) with intrathecal administration (3.3mg qd). Twelve days later, CRKP strains were still found positive in sputum and BALF culture but clean in cerebrospinal fluid. On day 35, the regiments for CRKP were changed to CAZ-AVI (2.5g q6h on the first day, then 2.5g q8h) and meropenem (1g q6h). The patient’s CrCl was 295.49 ml/min/1.73 m^2^ on day 35. Three days later, the TDM of CAZ-AVI results showed the following: CAZ C_max_ 57.13 mg/L and C_min_ 4.96 mg/L, and AVI C_max_ 21.267 mg/L and C_min_ 0.797 mg/L. On day 42, the BALF culture results showed that CRAB strains instead of CRKP strains were found after 7 days of CAZ-AVI treatment. So, tigecycline (TGC) (100mg q12h) combined with meropenem was used. However, CRKP was detected in BALF several times later. The patient’s infection failed to improve and he was transferred to a rehabilitation hospital for further treatment.

### Case 3

A 77-year-old man was hospitalized in NICU with progressive limb weakness and a temperature of 38.6°C on day 1. After admission, systemic examinations revealed pulmonary infection, hypertension, and chronic renal insufficiency. The initial anti-infective therapy was piperacillin/tazobactam. After 2 days, the patient was delirious, unresponsive, had low oxygen saturation, and was intubated. The inflammatory markers were high (PCT4.48 ng/ml, CRP 241 mg/L), and the antibacterial regiment was switched to meropenem 1g q8h. On day 23, the sputum and BALF culture results showed CRKP infection, which was just sensitive to CAZ-AVI (MIC = 4/4) and PMB (MIC≤1). The patient had renal insufficiency, and the CrCl was 39.78 ml/min/1.73 m^2^. CAZ-AVI (first day 2.5g q8h, later 1.25g q8h) combined with meropenem was used to anti-CRKP. TDM showed that CAZ C_max_ 77.22 mg/L, C_min_ 37.83 mg/L; AVI C_max_ 20.24 mg/L, C_min_ 8.67 mg/L. After 12 days of treatment with CAZ-AVI, the CRKP strains were clean in sputum and BALF culture, while the CRAB strains were isolated. The CAZ-AVI was replaced by PMB (75 mg loading dose with 50mg q12h maintenance dose) on day 40. On day 44, the patient died of gastrointestinal bleeding and lowered blood pressure.

### Case 4

A 35-year-old man had a poor appetite, fatigued for more than a month, and in coma for 20 days. On April 15, 2021, this patient was diagnosed with chronic and acute liver failure and hepatic encephalopathy. The patient was admitted to the department of liver transplantation on May 10, 2021. Under general anesthesia, the patient underwent orthotopic classical liver transplantation plus intestinal adhesiolysis. After surgery, the inflammatory markers were high (PCT 3.35 ng/ml, CRP 39.26 mg/L), and the patient was given the anti-infection treatment of meropenem (1g q8h), PMB (75mg q12h), teicoplanin (400mg qd), and caspofungin (50mg qd). On the 4th day of admission, CRKP (PMB MIC≤1, TGC MIC ≤ 1, CAZ-AVI MIC = 8/4) and CRAB were isolated from the sputum culture. Meropenem and teicoplanin were discontinued, and TGC (100mg q12h) combined with PMB was replaced to treat negative bacterial infection. Later, CRKP (PMB MIC ≤ 1, CAZ-AVI MIC = 8/4) was isolated from wound secretion, blood, and stool cultures. However, multiple sputum cultures, blood, and abdominal drainage fluid cultures indicated CRKP. On day 11 (2021.05.21), the CrCl of the patient was 170.44 ml/min/1.73 m^2^ and so TGC was replaced with CAZ-AVI 2.5g q8h. Three days later, the patient’s clinical infection symptoms and inflammatory indicators did not improve; considering ARC, CAZ-AVI was changed to q6h. Two days later, the TDM of CAZ-AVI results showed the following: CAZ C_max_ 111.18 mg/L and C_min_ 28.81 mg/L; and AVI C_max_ 32.95 mg/L and C_min_ 7.73 mg/L, while the CrCl decreased to 103.06 ml/min/1.73 m^2^. PMB was discontinued on day 17. On day 29, the patient’s PCT value decreased to 0.415 ng/ml and the CrCl was 100.29 ml/min/1.73 m^2^, while CRKP was still isolated from sputum. CAZ-AVI was given with 2.5g q8h to continue until day 39. Although the CRKP strains were still isolated from sputum on days 45 and 47, the patient had no fever with a slight cough and no sputum, and inflammatory markers tended to be normal. After 50 days of treatment, the patient was discharged.

### Summary of Cases

The basic information of the four cases is shown in Table 1. Patients were all men with ages ranging from 35 to 77 years. Three patients were admitted to NICU with neurological symptoms, and one patient was admitted to the liver transplantation department because of hepatic failure. All patients had HAP caused by CRKP during hospitalization. Case 1 also had UTI; Case 2 also had intracranial infection; Case 4 had BSI and ABSSSI, and all were infected with CRKP. MER + CAZ-AVI regimens were used for three patients used and CAZ-AVI + TGC regimens were used for one patient. Two patients showed good responses to the combined regimen of CAZ-AVI, and one patient died because of gastrointestinal bleeding and lowered blood pressure. The anti-infection effect of CAZ-AVI was not good in Case 2.

**TABLE 1:** Clinical characteristics of patients.

Parameters	Case 1	Case 2	Case 3	Case 4
Age (years)	56	32	77	35
Sex	Male	Male	Male	Male
Admission department	NICU	NICU	NICU	Liver Transplantation Department
Underlying disease	Hypertension, secondary epilepsy, liver insufficiency	Hypertension, arrhythmology, pneumonia	Hypertension, pneumonia, gout, old tuberculosis, hemifacial spasm	Liver failure, hepatic encephalopathy, chronic viral hepatitis B, spontaneous peritonitis, pneumonia
The highest temperature (°C)	39.5	39.3	39	40
WBC (normal 4–10, × 10^9^/L)	10.15	18.22	11.35	10.95
Neutrophil ratio (%)	82.2	94.5	82.5	90.4
CRP (mg/L)	44.9	278	241	168.4
PCT (ng/ml)	0.179	0.321	4.48	39.6
Infection type	HAP, UTI	Intracranial infection, HAP	HAP	BSI, HAP, ABSSSI
Treatment regimens for CRKP (days)	MER + CAZ-AVI (15)	MER + PMB; MER + CAZ-AVI (9)	MER + CAZ-AVI (12)	MER + PMB; MER + TGC; CAZ-AVI + TGC (28)
Outcomes	Effective	Ineffective	Dead	Effective

UTI, urinary tract infection; HAP, hospital acquired pneumonia; ABSSSI, acute bacterial skin and skin-structure infection; CAZ-AVI, ceftazidime-avibactam; TGC, tigecycline; MER, meropenem; CRKP, carbapenem-resistant *K. pneumoniae*.

All the CRKP strains produced KPC enzymes and were isolated from urine, sputum, BALF, cerebrospinal fluid, wound secretion, blood, and stool (Table 2). All strains are sensitive to PMB (MIC ≤ 1) and CAZ-AVI (MIC 2/4, 4/4, and 8/4). Most strains were intermediate sensitive to TGC, and all strains were resistant to CAZ (MIC > 32). After CAZ-AVI-based regimen treatment, CRKP was clean in Cases 1–3, and the blood of case 4.

**TABLE 2:** *In vitro* susceptibility of CRKP to the tested antibiotics (with MICs in μg/mL given in parentheses) and bacterial clearance.

Cases	Sources of CRKP isolated specimens	Carbapenemase	Bacterial clearance (days)	Antibiotics*			
				CAZ-AVI	PMB	TGC	CAZ
Case 1	Urine	KPC	Yes (8)	S (2/4)	S (≤1)	I (4)	R (>32)
	Sputum/BALF	KPC	Yes (9)	S (2/4)	S (≤1)	S (1), I (4)	R (>32)
Case 2	Cerebrospinal fluid	KPC	Yes (12)	S (2/4)	S (≤1)	I (4)	R (>32)
	Sputum	KPC	Yes (7)	S (2/4)	S (≤1)	I (4)	R (>32)
Case 3	Sputum/BALF	KPC	Yes (9)	S (4/4)	S (≤1)	I (4)	R (>32)
Case 4	Sputum	KPC	Not cleared	S (8/4)	S (≤1)	S (≤1)	R (>32)
	Wound secretion	KPC	No repeated testing	S (8/4)	S (≤1)	I (4)	R (>32)
	Blood	KPC	Yes (4)	S (8/4)	S (≤1)	I (4)	R (>32)
	Stool	KPC	No repeated testing	S (8/4)	S (≤1)	S (2)	R (>32)

CAZ-AVI, ceftazidime-avibactam; CAZ, ceftazidime; KPC, *K. pneumoniae* carbapenemase; PMB, polymyxin B; S, sensitive; I, intermediate; BALF, bronchoalveolar fluid.

* Resistant to all other antibiotics.

Cases 1 and 2 were ARC patients with CRCL 194.98 and 295.49 ml/min/1.73 m^2^. The AVI C_min_ was 3.16 mg/L when Case 1 was given CAZ-AVI 2.5g q6h. Moreover, in Case 2, after CAZ-AVI 2.5g q6h was given on the first day and 2.5q8h was given subsequently, the AVI C_min_ was only 0.797 mg/L, and the CAZ C_min_ was only 4.96 mg/L. Case 3 had renal insufficiency with CRCL 39.78 ml/min/1.73 m^2^, and TDM showed that the C_min_ of CAZ and AVI were higher than the MIC value after CAZ-AVI 2.5g q8h was given on the first day and 1.25 q8h was given subsequently. In case 4, the renal function was normal, and the plasma concentration of CAZ-AVI was higher than the MIC value after CAZ-AVI 2.5g q8h was given for 3 days and then 2.5g q6h for 2 days (Table 3).

**TABLE 3:** Renal function and blood concentration of CAZ-AVI detected by TDM.

Cases	CAZ-AVI regimen	CrCl (ml/min/1.73m^2^)	CAZ C_max_ (mg/L)	CAZ C_min_ (mg/L)	AVI C_max_ (mg/L)	AVI C_min_ (mg/L)
Case 1	2.5g q6h	194.98	149.21	14.33	37.76	3.16
Case 2	2.5g q6h first day, then 2.5q8h	295.49	57.13	4.96	21.267	0.797
Case 3	2.5g q8h first day, then 1.25 q8h	39.78	77.22	37.83	20.25	8.67
Case 4	2.5g q8h for 3 days, then 2.5g q6h for 2 days to TDM	Before CAZ-AVI use: 170.44, TDM day: 103.06	111.18	28.81	32.95	7.73

CrCl, creatinine clearance; CAZ, ceftazidime; AVI, avibactam; TDM, therapeutic drug monitoring.

## Discussion

This report described the therapeutic drug monitoring of CAZ-AVI concentrations in *K. pneumoniae* carbapenemase (KPC)-producing CRKP-infected patients with different kidney statuses, such as augmented renal clearance (ARC), normal renal function, and renal insufficiency. TDM results demonstrated that CAZ-AVI steady-state plasma concentration varied among patients with different kidney statuses, providing evidence for the utility of TDM of CAZ-AVI in individualized drug dose adjustment.

CAZ and AVI have similar pharmacokinetics and both are eliminated mainly through kidneys (Merdjan et al., 2015). Therefore, the renal function status significantly affects the PK/PD of CAZ-AV, thus influencing the clinical and microbiological efficacy (Merdjan et al., 2017).

Augmented renal clearance (ARC) is a pathophysiological condition defined as the occurrence of a measured CrCl ≥130 ml/min/1.73 m^2^ in males and ≥120 in females coupled with a normal serum creatinine value (0.6–1.4 mg/dl) (Hobbs et al., 2015; Cook and Hatton-Kolpek, 2019). ARC occurred in patients with subarachnoid hemorrhage, trauma, burn, and sepsis patients. A subgroup analysis of the REPROVE trial about CAZ-AVI showed that in ARC patients, the standard dosage of 2.5g q8h over 2 h ensured >95% probability of target attainment of a more conservative target of 50% fT > MIC, while a 35% decrease in drug exposure was observed compared to patients with normal renal function (mean AUC) (Das et al., 2019; Li et al., 2020). Real-world experience is limited to CAZ-AVI’s PK/PD analysis in two ARC critically ill patients. Treatment with 2.5g q8h over 2 h allowed the achievement of the conservative target of 50% fT > MIC against pathogens with a MIC up to 16 mg/L (Stein et al., 2019). For multidrug-resistant bacterial infection or severe infection, the higher the time-dependent antibacterial agents % fT > MIC, the better the efficacy is, and even the % fT > 4–5 × MIC is better (Stein et al., 2019). Furthermore, antibiotic dosing regimens required to suppress the emergence of resistance should be focused on achieving the PK/PD target of 100% fT > 4–8 × MIC (Guilhaumou et al., 2019). Previous studies demonstrated that the PK/PD targets for CAZ-AVI (CAZ 50% fT > MIC 8 mg/L and AVI 50% fT > C_T_ of 1 mg/L) were therefore deemed appropriate for the probability of target attainment (PTA) analyses to select ceftazidime-avibactam dosage (Coleman et al., 2014; Berkhout et al., 2016; Nichols et al., 2018; Das et al., 2019). A study used a 10,000-patient Monte Carlo simulation (MCS) to calculate the PTA and the cumulative fraction of response (CFR) for different CAZ-AVI dosage regimens to evaluate their efficacies and optimize the best initial dosage regimen found that for patients with CrCl values of 121–190 ml/min, and the standard dose (2.5g iv q8h) failed to reach 90% CFR against some *Enterobacteriaceae* members and *P. aeruginosa* (Dai et al., 2021). In the two patients with ARC, case 1 used CAZ-AVI 2.5g q6h, and AVI C_min_ was 3.16 mg/L. Case 2 used 2.5q8h (first day 2.5g q6h), and AVI C_min_ was only 0.797 mg/L. It implies that ARC patients may need more CAZ-AVI daily doses than the standard dose.

Due to the predominantly renal excretion of both CAZ and AVI, the concentrations of both drugs increase with increasing severity of renal impairment (Merdjan et al., 2017; Das et al., 2019). CAZ-AVI dosage adjustments are required according to different severities of renal insufficiency to minimize the risk of overexposure in patients with a CrCl of ≤50 ml/min (Li et al., 2020). The modified dosage regimens are now included in product labels, which advise close monitoring of CrCl in patients with renal impairment (Das et al., 2019). A study also discussed that patients with rapidly improving renal function without a timely change in dosage were underdosed (Li et al., 2020). PTAs for the CAZ-AVI original dosage regimens given to patients with moderate renal impairment in the event of renal function improvement to normal renal function were well below the targeted range (Li et al., 2020). Hence, if the renal insufficiency is caused by infection, the dose of CAZ-AVI can be appropriately increased according to the adjustment of the manual to control the infection better, and the dose can be adjusted timely with the improvement of renal function. Therefore, despite renal insufficiency after infection in patient 3, the creatinine clearance rate was 39.78 ml/min/1.73 m^2.^ The dose of CAZ-AVI given was standard on the first day and was adjusted to 1.25g q8h later. TDM results showed that the C_min_ values of CAZ and AVI were higher than the target concentration, and the PK/PD target rate was 100%.

In recent years, the extensive use of carbapenems increased carbapenem resistance in *K. pneumoniae*. The primary mechanism is the production of a carbapenemase classified into the Ambler class A (*K. pneumoniae* carbapenemase, KPC), B (the metalloenzymes NDM, VIM, and IMP), and D (oxacillin enzyme, OXA) (Logan and Weinstein, 2017). A previous study found that *KPC-2* was the most frequently detected gene in CRKP strains in Southwestern China (Tian et al., 2019). The coding gene of KPC is *blaKPC*, which can be transferred by the plasmid among *K. pneumoniae* strains through Tn3-based transposon Tn4401 (Logan and Weinstein, 2017). NDM-1 *K. pneumoniae* is a newly emerged highly resistant bacteria that can produce NDM-1, which is capable of breaking down beta-lactam antibiotics, including avibactam (Kumarasamy et al., 2010). To date, NDM-1 *K. pneumoniae* has also been reported worldwide, including in China (Tian et al., 2019).

We explored the use of TDM to evaluate the concentration and efficacy of CAZ-AVI in CRKP-infected patients with different kidney statuses. The limited clinical samples limit the results of this study. Large-scale clinical sample experiments are needed to confirm the formulation of an individualized administration scheme of CAZ-AVI based on different renal functions.

## Conclusion

In conclusion, our study demonstrated that CAZ-AVI steady-state plasma concentration varies among patients with different kidney statuses, including ARC, normal renal function, and renal insufficiency, providing evidence for the utility of TDM of CAZ-AVI in individualized drug dose adjustment. ARC patients may need more CAZ-AVI daily doses than the standard dose.

## Data Availability

The original contributions presented in the study are included in the article/Supplementary Material; further inquiries can be directed to the corresponding author.
